# Diagnostic bias during the COVID-19 era: COVID-19 or renal abscess?

**DOI:** 10.1177/0391560321993592

**Published:** 2021-08

**Authors:** Marco Amato, Ahmed Eissa, Giuseppe Rosiello, Rui Farinha, Pietro Piazza, Maria Chiara Sighinolfi, Bernardo Rocco, Giampaolo Bianchi, Salvatore Micali, Alexandre Mottrie, Stefano Puliatti

**Affiliations:** 1 Department of Urology, University of Modena and Reggio Emilia, Modena, Italy; 2 Department of Urology, OLV, Aalst, Belgium; 3 ORSI Academy, Melle, Belgium; 4 Urology Department, Faculty of Medicine, Tanta University, Tanta, Egypt; 5 Division of Oncology/Unit of Urology, URI, IRCCS Ospedale San Raffaele, Milan, Italy; 6 Vita-Salute San Raffaele University, Milan, Italy; 7 Department of Urology, University of Bologna, Bologna, Italy

**Keywords:** COVID-19, renal abscess, kidney tumor, Sars-CoV-2, diagnosis

## Abstract

**Introduction:**

The Coronavirus disease-2019 (COVID-19) has been declared as a pandemic in March 2020 by the World Health Organization (WHO). Since then, this pandemic has dramatically affected the entire world, even radically influencing the way patients are framed at triage. Symptoms and tests in most cases lead to a correct diagnosis; however, error may be around the corner.

**Case report:**

A 60 years old patient was referred with weight loss, fatigue and mild fever for 3 weeks as he was working in a COVID-19 ward. After a positive swab and chest CT scan, he was admitted in the hospital and treated as mild COVID-19 patient. A CT scan performed after the patient was discharged revealed a renal lesion misidentified as a tumor then clarified to be an abscess which retrospectively appears to be the main cause of his symptoms.

**Conclusion:**

Clinicians should consider other life-threatening disease in the differential diagnosis of patients presenting with similar symptoms to minimize mistakes and avoid further unnecessary investigations.

## Introduction

Coronavirus disease-2019 (COVID-19) pandemic has spread rapidly all over the world, putting COVID-19 in the spotlight, being the “pathology of the hour.” With approximately 20 million positive cases and 730 thousand deaths, all national healthcare systems were put to the test.^1,2^

The clinical spectrum can vary from mild symptoms (cough and loss of taste and smell) to severe respiratory failure. Even with mild symptoms, our level of suspicion should be high in risky groups.^3–5^ Noteworthy, symptoms alone may be deceiving, and other life-threatening pathologies should not be underestimated, even in times of a pandemic where generalized fear can be deceptive. We present a case report of a 60-year old patient, admitted to the hospital with mild respiratory symptoms, fever and COVID-19 positive swab treated as SARS-CoV-2, who was found to have another potentially life-threatening disease.

## Case report

**Figure 1.**
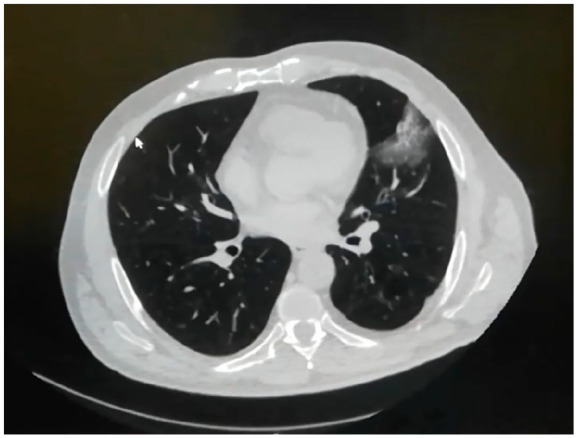
Thorax CT scan: left apical interstitial pneumonia.

**Figure 2.**
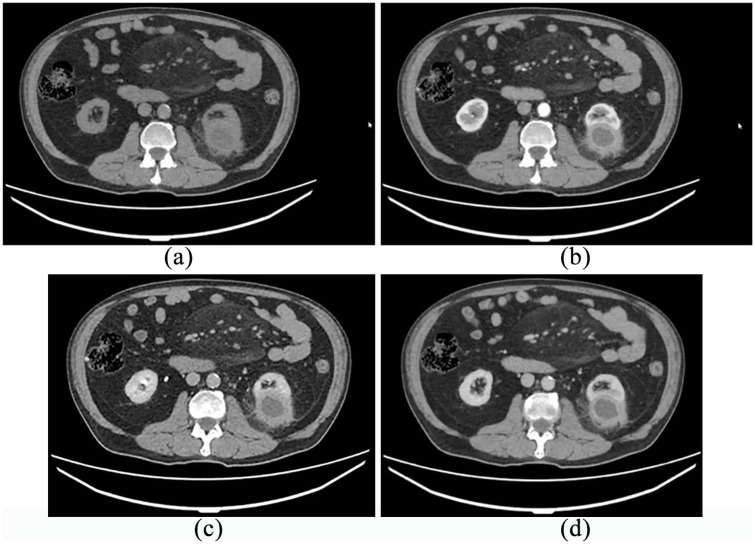
Abdominal CT scan: (a) no contrast, (b) arteriosus phase, (c) venous phase, and (d) excretory phase.

**Figure 3.**
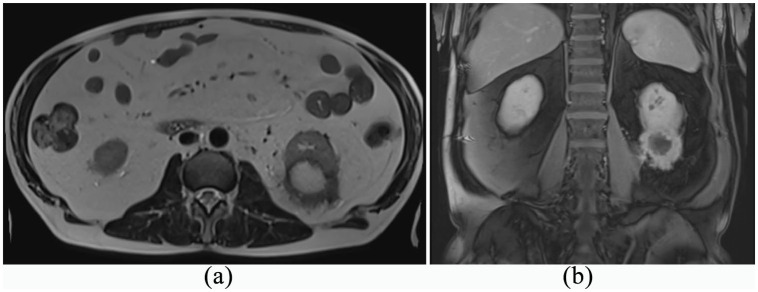
Abdominal MRI: (a) T1-weighted axial image and (b) coronal image.

A 60-year-old diabetic man, with a history of regular medical follow-up, was admitted to the hospital by the beginning of March 2020. Mild fever, malaise, and weight loss occurred about 3 weeks before hospitalization without any other symptoms. As the patient was an anesthetist working in an Italian COVID-19 ward, and complying with a strict testing protocol, he had two negative COVID-19 swabs in the last weeks. However, due to the persistence of general malaise and one episode of fever, he had a third swab that demonstrated positive results for COVID-19. A chest CT scan showed left basal interstitial pneumonia (Figure 1), so the patient was hospitalized with COVID-19 diagnosis. At admission his vital signs were stable. On physical examination there was abdominal distension and rebounding pain on the left upper quadrant of the abdomen. Laboratory tests are summarized in Table 1. Being labeled as “COVID-19 positive” patient, he received intravenous ceftriaxone and clarithromycin, hydroxychloroquine, enoxaparin, vitamin D and tocilizumab for 2 weeks. During the hospital stay the patient remained stable showing no exacerbation of respiratory symptoms (minimum O2ps 94%) but only mild fever for three consecutive days. No further medications were administered nor support with oxygen was needed. After 15 days and two consecutive negative swabs the patient was discharged. The last blood tests are showed in the Table 1. A CT scan was performed 2 days after discharge (Figure 2) showed persistent interstitial pneumonia and also an incidental unclear shape of the left kidney, that motivated a third thoraco-abdomin-pelvic CT scan. The total body CT showed a 5.4 × 4.5 cm mass with thick walls and central colliquation, involving the renal parenchyma and the renal fat but without involving the renal sinus, suspicious for advanced malignant tumor. A previous CT done 1 year earlier for other purpose (diverticulitis) did not show any pathology in the left kidney. An MRI scan was then performed to better clarify the nature of the lesion, but no certain diagnosis was achieved (Figure 3). After urological consultation done in another hospital, the initial decision was to perform radical nephrectomy for suspicious of an advanced malignant renal disease. However, after a second opinion of an expert, considering the reported symptoms previously attributed exclusively to the virus, renal abscess was suspected and a renal biopsy was recommended. This lesion was then drained and specimens were sent to cytologic examination showing it was a renal abscess (presence of inflammatory cells and no bacteria) which did not require surgical removal. The patient started oral antibiotic treatment and performed a follow-up ultrasound at 3 months that revealed reduction in the diameter of the renal abscess.

**Table 1.** Blood tests at 1st day and at 15th day.

Blood tests	Reference values	First day	15th day
Hemoglobin (g/dL)	13.5–17.5	16	15.4
Platelet	150–400 10^3^/uL	235 10^3^/uL	347 10^3^/uL
White blood cells	4.00–10.0 10^3^/uL	14 10^3^/uL	9 10^3^/uL
Neutrophils (%)	41–73	70	75
Lymphocytes (%)	19.4–44.9	21	12
Creatinine (mg/dL)	0.72–1.25	1.29	0.97
Hs-C-reactive protein (mg/dL)	<0.5	2.17	0.06
Glucose (mg/dL)	70–105	108	150
Procalcitonin (ng/mL)	0.00–0.08	0.05	–
Interleuchyn-6 (pg/mL)	<3.4	16.1	12.6
VES (mm)	0–20	37	12
Sars-CoV-2 IgM (AU/mL)	<10	42.03	21.92
Sars-CoV-2 IgG (AU/mL)	<10	71	67
Urine-culture	Negative	Negative	–

## Discussion

COVID-19 pandemic is an unprecedented medical emergency, where, the healthcare systems all over the world took extreme measures, stopping all non-urgent elective surgeries, increasing the financial support and the number of personal protective devices, and directing all the available medical resources to face this pandemic.^1^ Considering the highly contagious nature of SARS-COV-2, healthcare personnel were classified among the groups at high risk of getting infected as they are in close contact with confirmed COVID-19 patients.^6^ Furthermore, the most severe cases were surprisingly manifested in younger medical staff with fever and cough as main symptoms. This could be due to the longer working hours of younger personnel, but data to support this is not yet available.^7^ Importantly, the massive increase in the number of patients beyond the capacity of the healthcare systems and the increased risk of infection amongst the medical workforce result in workers burnout that can lead to error.^8^ Moreover, pitfall of judgment because COVID-19 is a more attractive diagnosis could direct the physician in the wrong direction.^9^

Renal abscess is a relatively uncommon debilitating and potentially fatal disease (1–10 in 10,000 hospital admissions) with mortality rates historically ranging from 12% to 50% and diabetes appears to be the leading risk factor. Fever and flank pain are usually the main symptoms, however nonspecific symptoms have been observed, such as weight loss, fatigue and rarely lower respiratory tract symptoms.^10^ Urinary tract infections are usually the cause of a renal abscess, however in our case both urine culture and aspirate were negative. In the literature according to our knowledge there are no cases of aseptic renal abscesses of unknown etiology. A case report was recently published on a renal abscess in a patient suffering from Chron disease, often associated with aseptic abscesses.^11^ In our case there is no pathology that can explain the onset of sterile abscesses. However, diabetes is the most important predisposing factor for renal abscesses which are generally infectious. We think that the bacterial load of the pathogen responsible for the renal abscess in our case has been eliminated by the use of broad-spectrum antibiotics according to the pharmacological COVID-19 protocol used. In the current clinical case, the symptoms were ambiguous, but positivity of the swab and the chest CT scan resulted in the underestimations of other symptoms that are more likely related to the abscess. However, it was reasonable to focus on COVID-19 because of history, other main symptoms and work category risk that have been misleading. Moreover, in the first instance according to CT and MRI images and negative urine culture, malignancy of the lesion suitable for nephrectomy was the main hypothesis and not abscess, but only kidney biopsy unveiled the real nature of the lesion sparing an invasive surgery.^12^ On the other hand, we cannot exclude the overlapping of two pathologies at the same time.^13,14^ Acute renal injuries from COVID have been described in the literature,^1^ but no cases of virus-related abscess. However, the impact on immune system due to COVID can be a predisposing factor for infectious diseases.^15^ In the same setting, the absence of symptoms and fully recovery of blood tests even with CT signs of pneumonia suggests more for abscess-related initial illness rather than COVID. This case must be contextualized in the historical moment of maximum criticality for the healthcare systems.^16^ The fear of contagion, the harmful consequences of the COVID-19 and the small knowledge at that time, added to the subsequent fear of a potential advanced tumor were smoke and mirrors for clinicians who have over diagnosed and overtreated the patient.

## Conclusion

COVID 19 is a tremendous problem that is affecting the economic system and the health system around the world, however overdiagnosis can lead to error. Other pathologies can have symptoms similar to moderate forms of COVID and accurate examination should be carried out before running to conclusion. This is an example of how a kidney disease can mislead the clinician for pitfall of judgment.
